# Highlight report: Erroneous sample annotation in a high fraction of publicly available genome-wide expression datasets

**DOI:** 10.17179/excli2015-760

**Published:** 2015-12-21

**Authors:** Marianna Grinberg

**Affiliations:** 1 Department of Statistics, TU Dortmund University, 44139 Dortmund, Germany

## ⁯⁯

Recently, Lohr et al. have published a method that identifies sample annotation errors in gene expression data (Lohr et al., 2015[18]). Surprisingly, 40 % of 45 analyzed publicly available datasets including 4913 patients were affected by erroneous sample annotation. The authors conclude that sample annotation errors may be a more widespread phenomenon as previously expected (Lohr et al., 2015[18]). The authors used two strategies for identifying sample mix-up. First, a classifier was established that differentiates between samples from female and male patients. This classifier is based on the x-chromosomal gene XIST and the y-chromosomal genes RPS4Y1 and DDX3Y (Lohr et al., 2015[18]). In datasets with similar numbers of male and females, approximately half of sample mix-ups will result in sex mislabeling. A further possible error is sample duplication, where the same sample is analyzed twice and the duplicate is erroneously labeled with another patient (Lohr et al., 2015[18]). To identify such duplications, a correlation-based strategy was used. A strength of the techniques presented by Lohr et al. is that they include normalization steps which make it possible to apply the same algorithm on samples of all datasets. The algorithm then differentiates between 'correctly classified' and 'misclassified' samples. In the analyzed 45 publicly available cohorts 18 contained at least one misclassification. The authors also show that deleting the erroneous samples can strongly influence the number of statistically significant prognostic genes.

Currently, genome-wide data are frequently used in cancer research (Stock et al., 2015[33]; Sicking et al., 2014[28]; Cadenas et al., 2014[4]; Mattson et al., 2015[21]). Intensively studied fields are breast- and ovarian cancer (Siggelkow et al., 2012[29]; Godoy et al., 2014[11]; Stewart et al., 2012[31]; Schmidt et al., 2012[24]). It can be expected that cohorts with only samples from either females or males have a lower risk of sex mislabeling. Therefore, it was surprising that an example of mislabeled patients was also identified in breast cancer patients. For example the well-known TRANSBIG cohort contains one female node-negative breast cancer patient who in reality is a man (Lohr et al., 2015[18]).

Besides its intensive use in cancer research (Micke et al., 2014[22]; Schmidt et al., 2008[24]; Botling et al., 2013[3]) genome-wide expression data are also frequently used in toxicology (Campos et al., 2014[5]; Stöber et al., 2014[32]; Marchan, 2014[20][19]; Bolt, 2013[1]; Song et al., 2013[30]; Godoy et al., 2013[12]; Bolt et al., 2010[2]). The goal of these studies is to obtain first evidence of the mechanism of action of chemicals (Glahn et al., 2008[10]; Shimada et al., 2010[26]; Dika Nguea et al., 2008[6]; Hendrickx et al., 2014[15]; Shinde et al., 2015[27]; Yao and Costa, 2014[34]; Gagné et al., 2013[9]; Fang et al., 2014[8]; Kim et al., 2012[16]) or to establish classifiers of co-regulated gene clusters (Grinberg et al., 2014[14]; Shao et al., 2014[25]; Krug et al., 2013[17]; Godoy et al., 2015[13]; Rempel et al., 2015[23]; Doktorova et al., 2012[7]). In these datasets the correlation-based classifier for sample duplication may be helpful. In conclusion, the easy to use classifiers published by Lohr and colleagues (2015[18]) should be routinely included into the analysis of gene array but also RNA seq data to reduce the number of erroneous sample annotations.
